# Increasing Utilization of Scrubbing Devices for Peripheral Intravenous Disinfectable Needleless Closed Connectors

**DOI:** 10.7759/cureus.61662

**Published:** 2024-06-04

**Authors:** Vikasni Mohan, Joni M Maga, Marianfeli C Landino-Delgado, Lauren M Rouse, Richard H Epstein, Eva L O'Brien, Alecia L Stein, Danielle B Horn

**Affiliations:** 1 Anesthesiology, Perioperative Medicine and Pain Management, University of Miami Miller School of Medicine, Miami, USA; 2 Anesthesiology, Jackson Health System Center for Patient Safety, University of Miami Miller School of Medicine, Miami, USA

**Keywords:** anesthesiology, quality improvement, guideline adherence, needleless closed connector, disinfection, infection prevention and control

## Abstract

Introduction

Peripheral intravenous (IV) administration sets are a source of infection that increases morbidity, mortality, and healthcare costs. In this quality improvement project, we aimed to enhance compliance with peripheral IV hub disinfection at anesthesia induction to follow the American Society of Anesthesiologists (ASA) safe medication injection guidelines.

Methods

This study was conducted in the main operating suite of the University of Miami’s principal hospital between June and October 2023. Audits of scrubbing device utilization by the anesthesiology team and focus groups were conducted before and after two educational interventions. Educational efforts focused on increasing compliance with peripheral IV disinfection using scrubbing devices.

Results

Mean use per case, inferred from the number of devices dispensed, nearly doubled from 0.44 (95% CI, 0.37 to 0.59) to 0.82 (95% CI, 0.77 to 0.88) (P < 0.0001). Implications regarding steps to further enhance compliance are discussed.

Conclusions

Through a simple educational program, scrubbing device utilization increased significantly from baseline.

## Introduction

Peripheral intravenous (IV) administration sets can be portals to bloodstream infections, increasing morbidity, mortality, and healthcare costs when not disinfected [1-4]. Contamination can occur from inadequate disinfection of the hands, disinfectable needleless closed connectors (DNCCs), and syringe tips [5-7]. Despite the low incidence of peripheral IV-related bloodstream infections (0.2%), an estimated 51.4 million patients undergo inpatient surgery per year in the United States of America, the vast majority of them receiving a peripheral IV as part of their perioperative care [1,8]. This justifies the need for increased disinfection education to improve patient safety.

During induction of general anesthesia, there are multiple DNCC accesses, making disinfection especially important. The American Society of Anesthesiologists (ASA) guidelines recommend DNCC disinfection before medication administration [9]. Major challenges to compliance include the increased time required for disinfection while administering multiple time-sensitive bolus injections during induction. Other barriers to compliance include high task density, a lack of understanding of guidelines, poor habit formation, absence of visual reminders, and a work culture that does not prioritize infection control [10,11].

A single educational intervention has been shown to be as effective as a combination of strategies for improving compliance with disinfection protocols, especially when combined with feedback from an audit [12]. Improving clarification on one’s role in following disinfection protocol and informing individuals of the risks associated with nonadherence to guidelines were shown to improve behavioral change surrounding disinfection guideline compliance [13]. Site-Scrub® Isopropyl Alcohol devices (“scrubbing devices”) have multiple individual sponges saturated with 70% isopropyl alcohol, increasing friction and surface area, thereby offering superior decontamination of DNCC and hubs compared to alcohol swabs [14,15]. At the study hospital, such scrubbing devices were already stocked in each anesthesia machine. Despite the ready access to these devices and departmental guidelines, there was perceived low compliance with the existing protocols. This QI initiative was designed to educate anesthesia practitioners about the benefits of DNCC disinfection, to learn the barriers to compliance with the existing protocols, and to standardize procedures. Utilization of the scrubbing devices was selected as a surrogate measure to assess the success of the project, which was intended to be an initial step to improve adherence to safe injection practice guidelines [9].

## Materials and methods

The University of Miami Institutional Review Board exempted this study from written informed consent (#20231182; November 6, 2023). This manuscript adheres to the Enhancing the Quality of and Transparency of Health Research (EQUATOR) guidelines [16].

**Figure 1:**
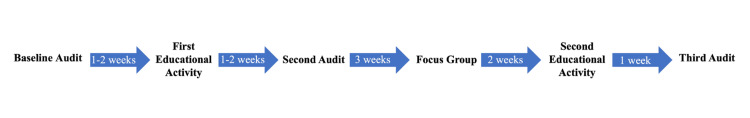
Timeline of the quality improvement study A baseline audit was performed over a five-day period to measure scrubbing device usage at the study hospital. An educational activity focused on compliance with scrubbing device use was conducted, followed by a second audit. A focus group was held thereafter, followed by a second educational activity, and then a third audit was undertaken to assess scrubbing device use. Image credit: Danielle Horn.

The main operating room (OR) suite of the University of Miami (a tertiary care medical center) comprises 17 ORs in which a diverse variety of inpatient and ambulatory surgical procedures are performed, with the exception of obstetrics and trauma surgery. From June 2023 to October 2023, we conducted a quality improvement (QI) initiative in this suite to improve compliance with IV hub disinfection, consisting of audits, educational activities, and focus groups (Figure 1). Anesthesiologists, anesthesiology residents, certified registered nurse anesthetists (CRNAs), and student registered nurse anesthetists (SRNAs) were included. However, only anesthesiologists and CRNAs were included in focus groups as they permanently staff these ORs, whereas SRNAs and residents temporarily rotate for one month at a time and make up a small fraction of the workforce.

Three audits measured scrubbing device usage over three separate five-day intervals, at baseline and after each of two training sessions. On the starting day of each interval, the supply of scrubbing devices was stocked to a par level of 30. On subsequent days, before the first case in each OR, the remaining scrubbing devices from the previous day were collected, and 30 new scrubbing devices were placed in the top drawer of each anesthesia machine. The stocking level greatly exceeded the number of cases performed daily in each OR, and scrubbing devices were not restocked during the day. The number of scrubbing devices utilized during the previous day was calculated as 30 minus the number of devices remaining. Staff were not informed that usage was being audited to mitigate a potential Hawthorne effect. 

**Figure 2:**
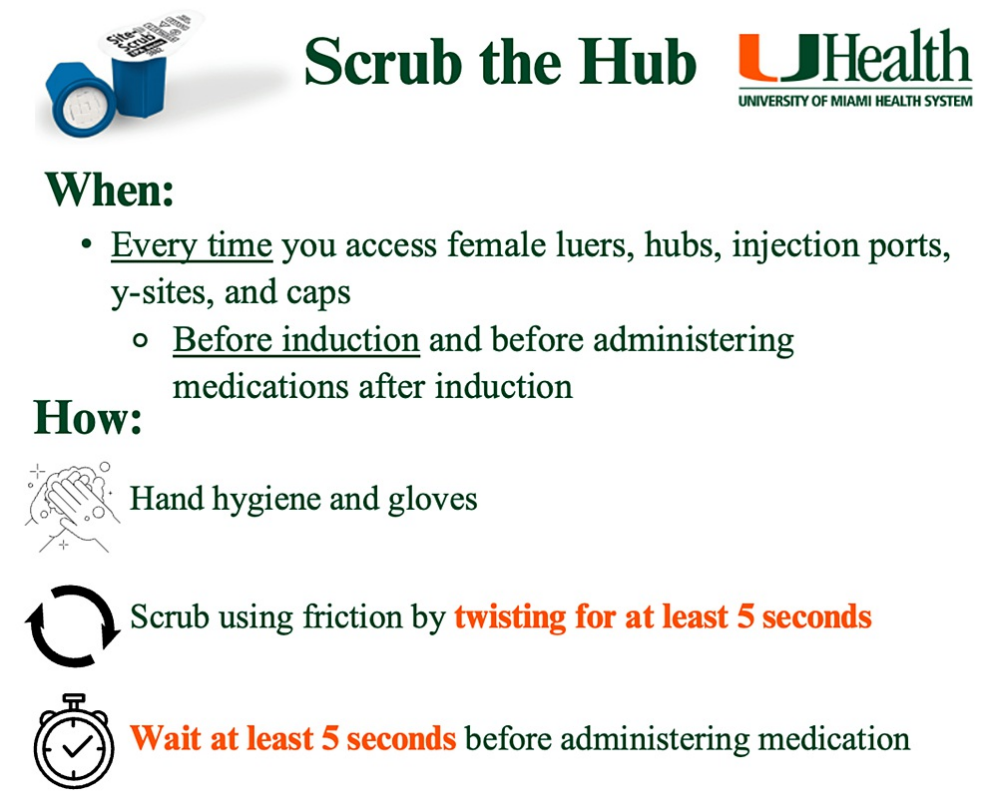
Infographic provided during the first educational activity Participants including anesthesiologists, anesthesiology residents, certified registered nurse anesthetists (CRNAs), and student registered nurse anesthetists (SRNAs) working at the study hospital between June and October 2023 were provided with this infographic during the first educational activity. This teaching session focused on the importance of compliance with scrubbing device use for peripheral IV disinfection. Image credit: Lauren Rouse.

**Figure 3:**
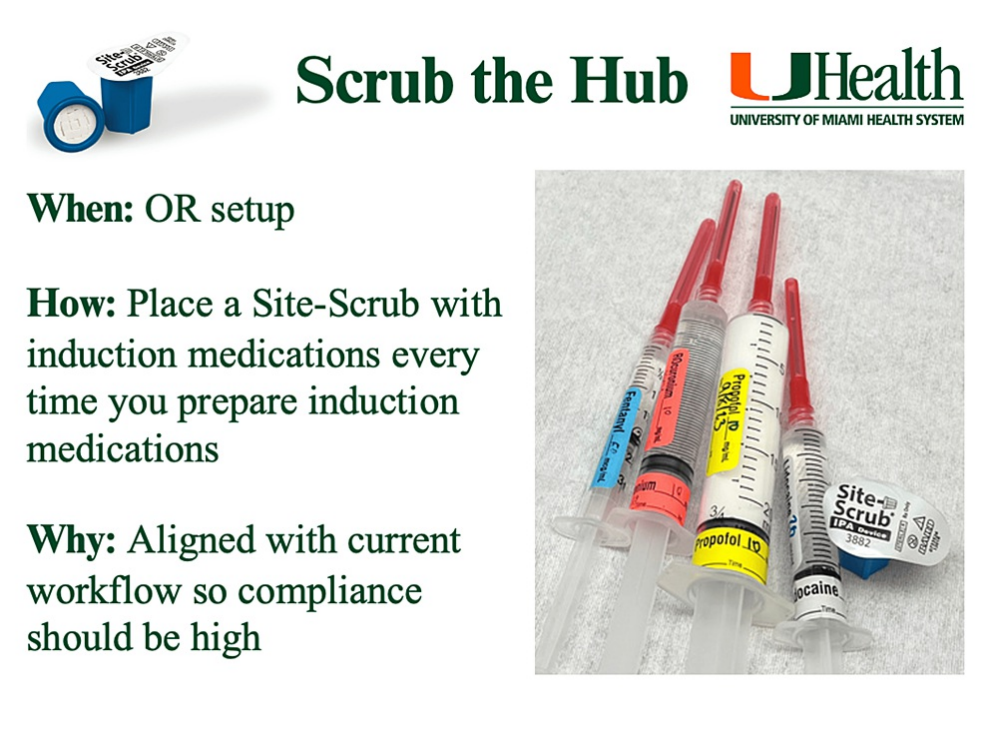
Infographic provided during the second educational activity Participants including anesthesiologists, anesthesiology residents, certified registered nurse anesthetists (CRNAs), and student registered nurse anesthetists (SRNAs) working at the study hospital between June and October 2023 were provided with this infographic during the second educational activity. Similar to the first session, this second activity focused on the importance of compliance with scrubbing device use for peripheral IV disinfection. Image credit: Lauren Rouse.

Two educational activities were conducted to increase compliance with ASA guidelines for peripheral IV disinfection. These activities emphasized the risks of bloodstream infections and encouraged proper scrubbing device usage (Figures 2, 3). The presentations were delivered in person, via Zoom (Zoom Video Communications Inc., San Jose, CA, USA), and through email and text message. Attendance was confirmed based on sign-in to ensure that staff were exposed to the materials. These educational materials were added to the existing orientation and onboarding materials for new staff joining the anesthesiology department at the study hospital. Between the two educational efforts, focus groups were held with CRNAs and attendings to address perceived barriers to compliance and to solicit recommendations for process improvement through surveys and open-ended questions. An infographic illustrating how the scrubbing device could be incorporated into the existing induction medication preparation process was created (Figure 3).

The number of scrubbing devices counted in the audit from each OR was divided by the total number of cases performed in that OR on the previous day to calculate the fraction of cases where the device was used for induction, assuming that a single scrubbing device was used at the start of each case. We presumed that scrubbing devices were neither discarded without use nor used following induction because such behavior was atypical based on previous observation. That is, the existing departmental practice was not to rescrub the hubs intraoperatively, and unopened devices were rarely seen in the trash containers. Qualitative data was collected through anonymous virtual polling and focus groups as part of routinely scheduled faculty and staff meetings.

The *prtest* module from Stata v17.0 (StatCorp, College Station, TX, USA) was used to compare the fractions of cases in each OR on each day between baseline and the educational interventions where a scrubbing device was inferred to have been used. A P-value <0.05 was required to claim statistical significance.

## Results

During the two educational efforts, 38 distinct anesthesiology attendings, 16 anesthesiology residents, 55 CRNAs, and six SRNAs participated in the educational activities. We reached 100% participation of staff working during the audit based on cross-referencing the schedules with either in-person attendance or virtual replies. 

Use of the scrubbing devices increased from 0.44 per case (95% CI, 0.38 to 0.50) at baseline to 0.82 per case (95% CI, 0.77 to 0.87) following the initial educational effort (P < 0.0001, Table 1). Improvement persisted compared to baseline after the second educational effort (mean 0.87 per case; 95% CI, 0.83 to 0.92), but there was no further improvement following the second educational effort (P = 0.16).

**Table 1:** Change in scrubbing device use from baseline to after the first and second audits ^a^During each of the five-day audits, 17 operating rooms were in use (N = 85 OR•day combinations). ^b^Calculations were based on the assumption that one scrubbing device was used per case. Fractions were computed for each of the 85 OR•days and then averaged. CI, confidence interval; QI, quality improvement; OR, operating room

QI stage	Scrubbing devicesused (N)	Cases audited^a^ (N)	Fraction of cases with scrubbing device use (95% CI)^b^	P-value (vs. baseline)	P-value (vs. first education)
Baseline	95	217	0.44 (0.37-0.50)		
After first education	160	194	0.82 (0.77-0.88)	<0.0001	
After second education	194	222	0.87 (0.83-0.92)	<0.0001	0.16

**Figure 4:**
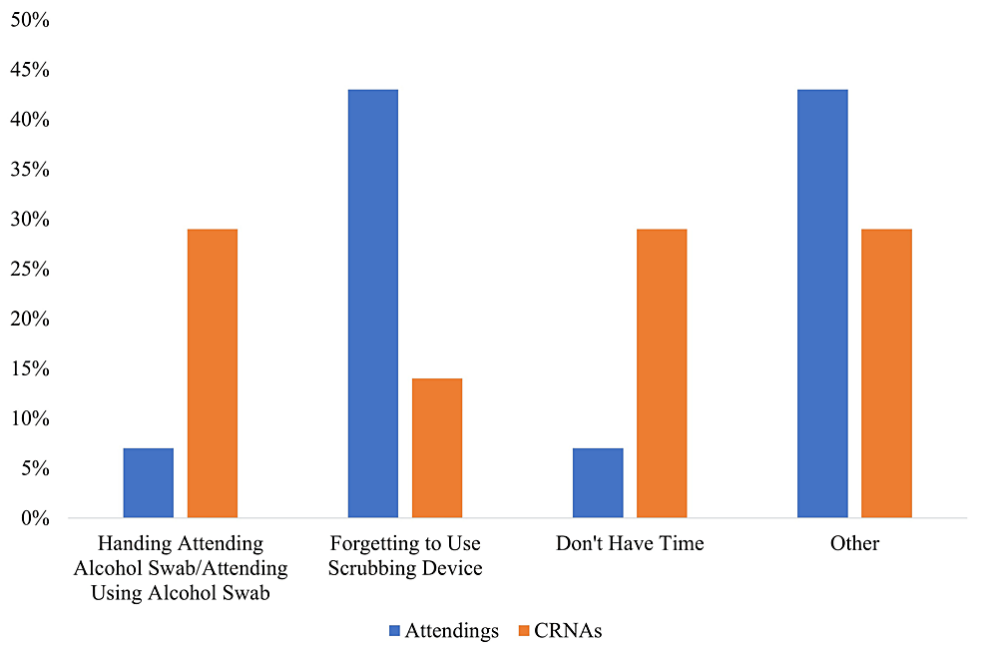
Staff reported reasons for not using scrubbing devices at induction There were 16 attendings and seven certified registered nurse anesthetists (CRNAs) who responded to the questionnaire. Image credit: Vikasni Mohan.

Analysis of feedback from the randomly selected focus groups (Table 2, Figure 4) revealed substantive differences between the perceptions of the CRNAs and the attendings. Whereas 71% of CRNAs responded that they “always” provide the attending with a scrubbing device before induction, 71% of attendings answered that they were “sometimes” provided with the scrubbing device. Furthermore, 57% of CRNAs responded that, if it was provided, the attending used the scrubbing device “sometimes,” whereas 79% of attendings responded that they “always” used it when provided. The CRNAs’ most common reasons for not providing the scrubbing devices were that they provided an alcohol swab instead (29%) or did not have time to provide the scrubbing device (29%), while attendings most often reported forgetfulness (43%).

**Table 2:** Focus group question responses CRNA, certified registered nurse anesthetist

Focus group questions	N	Always	Sometimes	Never
CRNAs				
Are you handing/providing the attending a scrubbing device before induction?	7	71%	29%	0%
For those who are consistently doing this, is the attending using it?	7	43%	57%	0%
Attendings				
Are the CRNAs handing you a scrubbing device before induction?	16	21%	71%	7%
For those of you who answered yes, how often are you using it before induction?	16	79%	14%	7%

## Discussion

Our limited education program was successful in increasing the utilization of scrubbing devices for peripheral IV DNCC disinfection. Barriers to compliance identified included a misunderstanding of the guidelines, time constraints, and the concealed location of the scrubbing devices (i.e., in a closed drawer of the anesthesia machine), similar to previous studies [17,18]. 

There was a substantive lack of concordance between the attending anesthesiologists and the CRNAs with respect to the process of providing and using the devices. This suggests a lack of communication between the two groups during induction regarding the device use. In some cases, the scrubbing devices were simply placed next to the syringes containing the induction drugs, and in other cases, there was a more direct interaction from the CRNA regarding use. However, during the focus group, we asked the attendings whether CRNAs were providing them the scrubbing device with the induction medications, while in the anonymous virtual poll, we asked whether the CRNAs were handing the attending a scrubbing device. This phrasing difference between the CRNA and attending focus groups may at least partially explain the discrepancy. In the future, interventions to improve communication between anesthesiologists and CRNAs can be implemented, including using closed-loop communication, participating in medical simulations involving practice scenarios, and addressing scrubbing device use during the anesthesia time out. Additionally, continued audits can assess the level of compliance going forward, and compliance challenges can be targeted by optimizing workplace practices to better integrate scrubbing device use and by creating a culture of safety that prioritizes adherence to safety measures.

This study’s primary limitation was inferring scrubbing device usage per case based on an accounting audit. Ideally, one would have an observer present (or a video recording) to allow determination if the device was used during induction and thereafter. However, the resources and financial support to perform such a study were unavailable, thus requiring our use of the surrogate measure related to the consumption of the devices. As a preliminary effort, had we not shown any increase in device utilization, we would have concluded that the educational process we employed was unsuccessful. However, the observation that utilization nearly doubled is encouraging, and this is significant because it demonstrated a measurable increase in scrubbing device use that can lead to better patient outcomes by reducing the risk of bloodstream infections. OR cases occurring during this study were primarily elective surgeries and did not include any trauma or obstetrical cases. Despite these limitations, these results are generalizable to similar anesthesiology departments based at academic medical centers where CRNAs, residents, and attendings work together to provide care for surgical patients. Our planned future efforts will be directed toward securing sufficient funding to directly measure the use of the scrubbing devices and to ensure that they are used according to the directions rather than conducting more audits as a surrogate measure of device consumption.

## Conclusions

This QI study demonstrated that a brief educational effort improved rates of scrubbing device use at an organization where scrubbing devices were readily available but where compliance was low. This effort represents an initial step toward achieving full compliance with the ASA guidelines for safe medication practices. Ongoing efforts include educational efforts to promote disinfection of the DNCCs before each medication administration during cases, improved machine disinfection during and after cases, avoiding medication preparation during the ongoing case for a subsequent case, and improving hand hygiene.
